# Appropriate use of anti-thrombotic therapy in patients with atrial fibrillation at single-center experience, Northwest Ethiopia

**DOI:** 10.1186/s12872-020-01659-y

**Published:** 2020-08-17

**Authors:** Ermiyas Endewunet, Abilo Tadesse, Aynishet Adane, Mohamed Abdulkadir

**Affiliations:** Department of Internal Medicine, College of Medicine and Health Sciences, University of Gondar, Gondar, Ethiopia

**Keywords:** Atrial fibrillation, Anti-thrombotic therapy, Northwest Ethiopia

## Abstract

**Background:**

Atrial fibrillation (AF) is the commonest clinically significant ECG-evidenced sustained cardiac arrhythmia in clinical practice. Disability and mortality attributed to AF is high in low-income regions like sub-Saharan Africa. The risk of stroke/TIA in patients with AF can be significantly reduced with anti-thrombotic therapy. Despite the existing evidence of its benefit, significant percentages of AF patients eligible for anti-thrombotic therapy are undertreated in the region.

**Methods:**

A hospital-based cross-sectional study was conducted to determine the appropriate use of anti-thrombotic therapy in patients with AF between December 1, 2018 and September 30, 2019 at Cardiac Clinic, University of Gondar hospital, Northwest Ethiopia. Consecutive sampling method was used to recruit 210 study subjects. Patients were interviewed to obtain socio-demographic data. Relevant medical history and laboratory parameters were obtained from patients’ records. Diagnosis of atrial fibrillation was based on detection of irregular arterial pulse and presence of ‘f’ waves on 12-lead ECG tracing. Clinical evaluation, echocardiography, chest X-ray and blood chemistry were used to diagnose underlying causes of AF. Data was entered into EPI Info version 4.4.1 and analyzed using SPSS version 20. Bi-variate and multi-variate logistic regression analyses were used to identify associated factors with appropriate use of anti-thrombotic therapy in patients with atrial fibrillation. *P*-values < 0.05 were used to declare significant association.

**Results:**

A total of 210 patients were included in the study. The mean age of patients was 51.29 ± 17.2 years. Two-thirds (145/210) of participants were females. Seventy-four (35%) had valvular AF, while 136/210 (65%) had non-valvular AF. Sixty-six percent (139/210) of study subjects were appropriately treated with anti-thrombotic therapy. Appropriately treated subjects in valvular AF group and non-valvular AF group were 58/74 (78%) and 81/136 (60%) respectively. On multi-variate analysis, ‘can afford for regular INR monitoring’ (AOR = 2.60 95% CI: 1.10–6.10, *P* = 0.001) was significantly associated with appropriate use of anti-thrombotic therapy.

**Conclusion:**

Sixty-six percent of AF patients eligible for anti-thrombotic therapy were appropriately treated. Intervention program to access ‘regular INR monitoring’ should be practiced to escalate utilization rate of anti-thrombotic therapy (warfarin) in eligible AF patients.

## Background

Atrial fibrillation (AF) is the commonest ECG-evidenced sustained cardiac arrhythmia in clinical practice. Although the burden of AF is greater in developed countries, disability and mortality attributed to AF is maximal in developing countries due to limited health care access [1–6]. Clinical risk factors (older age, hypertension, diabetes and heart disease), echocardiographic features (reduced left ventricular ejection fraction, left atrial enlargement, left atrial spontaneous echo contrast, complex aortic plaque), and cardiac biomarkers (increased serum high-sensitivity Troponins, N-Terminal pro-Brain Natriuretic Peptide) are associated with an increased risk of AF. Although rheumatic heart disease is the major cause of AF in sub-Saharan Africa, non-valvular cardiac causes are rising due to epidemiological transition in the region [5–8]. AF is responsible for 15–20% of all strokes, and causes 5-fold increased risk in ischemic stroke. Adjusted-dose vitamin K antagonists (warfarin) and antiplatelet agents reduce the incidence of stroke by 65 and 20% respectively [3–5, 9]. Guidelines recommend that non-valvular AF patients with CHA_2_DS_2_VASc score ≥ 2 and valvular AF patients regardless of stroke risk score should be started on antithrombotic therapy unless contraindicated [1–3, 9–12]. Despite the existing evidences and guidelines, several studies have demonstrated suboptimal use of oral anti-thrombotic therapy in clinical practice. Predictors of suboptimal use in eligible AF patients include, but not restricted to, female sex, elderly (age ≥ 75 years), rural residency, paroxysmal AF type, lower CHA_2_DS_2_VASc score, co-existing co-morbidity type, fear of bleeding complication, previous bleeding episodes, limited access to or high cost of INR monitoring, limited opportunity to implement guideline, and health care setting type [3–7, 9, 13–15].

Therefore, this study aimed to determine appropriate use of antithrombotic therapy in patients with AF in routine clinical practice in Northwest Ethiopia.

## Methods

### Study settings

A hospital-based cross sectional study was conducted between December 1, 2018 and September 30, 2019 at Cardiac Clinic, University of Gondar hospital. The hospital is located in Northwest Ethiopia, which is 750 km away from the capital, Addis Ababa. The hospital has a catchment population of 5 m people.

### Study subjects and variables

#### Study subjects

Patients with ECG-confirmed atrial fibrillation who had follow up at Cardiac Clinic, University of Gondar hospital, during the study period were considered as study population. Patients intolerant or contraindicated for anti-thrombotic therapy, and treated with anti-thrombotic therapy for other indications were excluded from the study.

#### Study variables

Dependent variables: Appropriate use of anti-thrombotic therapy in patients with AF.

Independent variables: 1) Socio demographic characteristics -age, sex, marital status, educational status and place of residence 2) Patient-related factors -eligibility for treatment, medication side effects, contra indication to treatment, and afford for INR monitoring 3) Clinical factors- heart failure, hypertension, diabetes, chronic kidney disease, CHA_2_DS_2_VASec score, type of AF, prior stroke/TIA, and hyperthyroidism.

#### Sample size and sampling procedure

The sample size was calculated using Fisher’s formula at a prevalence of 15% with a confidence interval of 95% and degree of precision of 5%. Consecutive sampling method was used to recruit 210 study subjects. No special events were noticed in study subjects or clinical care setting during the study period [16].

#### Data collection instrument and procedures

Data were collected through an investigator administered pre-designed questionnaire. Patients were interviewed to obtain socio-demographic data. Focused clinical examination was done to each of study subjects. Relevant medical history and laboratory parameters were obtained from patients’ records. Diagnosis of atrial fibrillation was based on detection of irregular arterial pulse and ‘f’ waves on 12-lead ECG tracing. Clinical evaluation, echocardiography, chest X-ray and blood chemistry were used to diagnose underlying causes of AF.

Twelve-Lead ECG (ECG 1200G, YSIP-155, Beijing, China) were performed on all patients by physician with standardization of 1 mV = 10 mm and paper speed of 25 mm/sec. ECG-based AF diagnoses were reviewed by a cardiologist.

Two-Dimensional Doppler Transthoracic Echocardiography (B/W Digital Ultrasound Scanner, ARI group, China) was performed for AF patients with heart failure by a cardiologist to determine abnormalities on ventricular ejection fraction, valve morphology, ventricular wall size and motion, and atrial and ventricular chamber dimensions.

Venous blood samples were collected from AF patients in plain tubes and centrifuged at 2500 rpm for 15 min at room temperature to obtain serum. Serum glucose and creatinine were determined by enzymatic glucose oxidase and kinetic alkaline picrate method respectively using Mindray BS-480 (Shenzhen Mindray Bio-Medical electronics Co., Ltd., China) clinical chemistry analyzer. Thyroid function tests (TSH, T_4_ and T_3_) were determined in whom thyroid disorders were suspected. Thyroid function tests were determined using Radioimmunoassay (RIA) technique (Roche, Switzerland), and kits were from Beijing Isotope Nuclear Electronic Co., Beijing, China.

#### Data analysis

Data were entered into EPI Info version 4.4.1 and transported to SPSS version 20 for analysis.

Patient characteristics were reported as counts (percentages) for categorical variables, and mean with standard deviation for continuous variables. Both bi-variate and multi-variate logistic regression analyses were used to identify independently associated factors with appropriate use of anti-thrombotic therapy in patients with atrial fibrillation. Those variables with a *P*-value < 0.2 in the bi-variate analysis were exported to multi-variate analysis to control the possible effect of confounders. Adjusted odds ratio (AOR) with 95% confidence interval (CI) and P-value < 0.05 were used to select independently associated variables with appropriate use of anti-thrombotic therapy in patients with atrial fibrillation.

#### Definition of terms

Atrial fibrillation: ECG-evidenced cardiac rhythm disorder where the normal atrial ‘P’ waves are replaced by chaotic, fibrillatory ‘f’ waves. It was clinically detected by irregular arterial pulse and confirmed by 12-lead ECG tracing [1, 2].

Anti-thrombotic therapy: Medications (anti-platelets and anticoagulants) which are given for patients with AF who are at high-risk of developing systemic embolic events.

Appropriateness to anti-thrombotic therapy: Patients are considered as appropriately treated with anti-thrombotic therapy when they are given oral anticoagulants for CHA_2_DS_2_VASc score ≥ 2, aspirin/oral anticoagulants for CHA_2_DS_2_VASc score of 1, no treatment for CHA_2_DS_2_VASc score of 0, oral anticoagulants for patients of valvular AF irrespective of the CHA_2_DS_2_VASc score, and oral anticoagulants for patients of hypertrophic cardiomyopathy with AF independent of the CHA_2_DS_2_VASc score. Patients with AF are inappropriately treated with anti-thrombotic therapy when they are not treated according to the above recommendation [1, 2].

CHA_2_DS_2_VASc score: Congestive heart failure or left ventricular ejection fraction < 40% (score 1), hypertension (score 1), age ≥ 75 years (score 2), diabetes (score 1), prior stroke/TIA (score 2), vascular diseases (prior myocardial infarction, peripheral arterial disease or aortic plaque) (score 1), age 65–74 years (score 1), sex category (female, score 1).

Framingham criteria: Clinical criteria for heart failure diagnosis, and require presence of either two major, or one major and two minor criteria. Major criteria include paroxysmal nocturnal dyspnea, neck vein distension, acute pulmonary edema, positive hepatojugular reflex, rales, S_3_ gallop, increased venous pressure > 16 cmH_2_0, cardiomegaly, and weight loss ≥4 kg in response to treatment. Minor criteria include nocturnal cough, dyspnea on ordinary exertion, pleural effusion, tachycardia (Pulse rate ≥ 120 bps), hepatomegaly, extremity edema, and vital capacity reduced by one-third from normal.

HAS-BLED score: Uncontrolled hypertension (score 1), abnormal kidney or liver disease (score 1, each), stroke (score 1), bleeding predisposition or tendency (score 1, each), labile INR (TTR < 60% score 1), elderly (age ≥ 65 years, score 1), and drugs or alcohol (score1, each). HAS-BLED score is used to assess the risk of bleeding, in which high risk patients (score ≥ 3) should be reviewed and followed frequently [17].

TTR (Time in INR Therapeutic Range): The duration of time in which the patient’s international normalized range (INR) values were within a desired range (INR = 2–3).

Valvular-AF: AF in the presence of moderate to severe mitral stenosis, mechanical prosthetic valve, or mitral valve repair.

Non-valvular AF: AF in the absence of moderate to severe mitral stenosis, mechanical prosthetic valve, or mitral valve repair.

Hypertension: Presence of persistently elevated systolic blood pressure ≥ 140 mmhg and/or diastolic blood pressure ≥ 90 mmhg in patients aged 18 years of age and above, history of hypertension, or the use of anti-hypertensive drug(s).

Diabetes mellitus: Fasting serum glucose ≥126 mg/dl, history of diabetes, or use medications for diabetes.

Chronic kidney disease: Abnormalities of kidney structure or function present for more than 3 months, with implications for health. Diagnosis of chronic kidney disease was settled by clinical, biochemical (raised serum creatinine) and/or imaging (ultrasound-proven reduced kidney size) findings.

Heart failure: Clinical syndrome that results from any structural or functional impairment of ventricular filling or ejection of blood. The Framingham criteria were used to diagnose heart failure.

Stroke/TIA: Neurological deficit attributed to an acute focal injury of the central nervous system by a vascular cause. Diagnosis of stroke/TIA was settled by clinical and imaging (brain CT/MRI) evaluation.

Hyperthyroidism: Clinical state that involves excess synthesis and secretion of thyroid hormones by the thyroid gland. Diagnosis of hyperthyroidism was made in the presence of suggestive clinical symptoms and signs including enlarged thyroid gland, and confirmed by RIA test from serum revealing low TSH and/or raised T_3_/T_4_.

#### Ethical considerations

Ethical clearance was obtained from the Institutional Review Board of College of Medicine and Health Sciences, University of Gondar. Formal letter of permission was obtained from University of Gondar hospital administrative body. Study subjects were recruited only after informed written consent was obtained from them. All data obtained were treated confidentially. During the data collection process, those patients who were found to have atrial fibrillation were taken care of as per the recommendations of AHA/ACC/HRS guidelines for the management of patients with atrial fibrillation.

## Result

### Socio demographic characteristics of study participants

A total of 210 patients with AF were included in the study. The male-to-female ratio was 1:2 (65 males and 145 females). The mean age of patients at diagnosis was 51.3 ± 17.2 years. Majority of them (75%) were aged less than 65 years. Half of them (51%) were rural residents and (56%) never joined formal education. Almost all of them were non-smoker (97%) and casual alcohol consumer (93%) (Table 1).

**Table 1:** Socio-demographic characteristics of patients with atrial fibrillation at Cardiac Clinic, UOG hospital, Northwest Ethiopia, December 1, 2018 to September 30, 2019

Variable	Number (No)	Percent (%)
Age category		
< 65 years	158	75.2
65–74 years	34	16.2
≥ 75 years	18	8.6
Sex		
Male	65	31.0
Female	145	69.0
Residence		
Rural	102	48.6
Urban	108	51.4
Marital status		
Married	149	71.0
Unmarried	61	29.0
Occupation		
Government employee	19	9.4
Merchant	20	9.5
Farmer	92	43.7
House wife	50	23.7
Student	13	6.1
Others*	16	7.6
Level of education		
Unable to read and write	118	56.2
Able to read and write	39	18.6
Primary education	17	8.1
Secondary Education	15	7.1
College and above	21	10.0
Monthly income (Eth. Birr)		
< 1500	130	61.9
1500–3000	49	23.3
> 3000	31	14.8

Note: * = retired or no job

### Clinical characteristics of patients

**Fig. 1:**
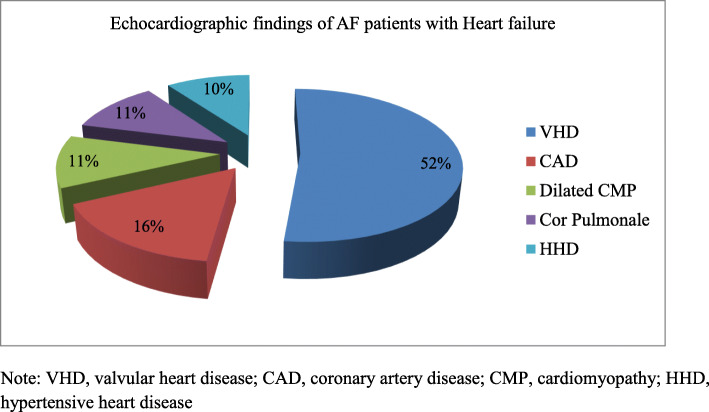
Echocardiographic findings of 191 AF patients with heart failure at Cardiac Clinic, UOG hospital, December1, 2018 to September 30, 2019

Seventy-four (35%) patients had valvular AF, predominated by rheumatic mitral valve disease, while 136/210 (65%) had non-valvular AF (Table 2). Heart failure (91%) was the commonest identified clinical condition in AF patients, followed by hypertension (22%), stroke/TIA (19%), and hyperthyroidism (18%) (Table 3). Underlying causes of heart failure were valvular heart disease (52%), coronary artery disease (16%), dilated cardiomyopathy (11%), Cor pulmonale (11%) and hypertensive heart disease (10%) (Fig. 1). The CHA_2_DS_2_VASc scores of patients with non-valvular AF were calculated and most (96%) of them scored 2 or above.

**Table 2:** Type of AF among patients with AF at Cardiac Clinic, UOG hospital, Northwest Ethiopia, December 1, 2018 to September 30, 2019

Type of AF	Number (No)	Percent (%)
Non-valvular AF	136	64.8%
CHA_2_DS_2_VASc score		
Female	1	0.7
1	4	2.9
2	70	51.5
3	28	20.6
4	23	16.9
5	10	7.4
Valvular AF	74	35.2%
Mitral stenosis (moder-Severe)	69	93.2
Mitral valve repair	2	2.7
Prosthetic valve (Mechanical)	3	4.1
Total	210	100

**Table 3:** Clinical characteristics of patients with atrial fibrillation at Cardiac Clinic, UOG hospital, Northwest Ethiopia, December 1, 2018 to September 30, 2019

Variables	Number (No)	Percent (%)
Smoking		
Yes	7	3.3
Never	203	96.7
Alcohol intake		
Never	88	41.9
Occasionally	108	51.4
Usually	14	6.7
Hypertension		
Yes	47	22.4
No	163	77.6
Heart failure		
Yes	190	90.5
No	20	9.5
Diabetes mellitus		
Yes	6	2.9
No	204	97.1
Stroke/TIA		
Yes	39	18.6
No	171	81.4
Hyperthyroidism		
Yes	38	18.1
No	172	81.9
Chronic kidney disease		
Yes	17	8.1
No	193	91.9

### Anti-thrombotic therapy

**Fig. 2:**
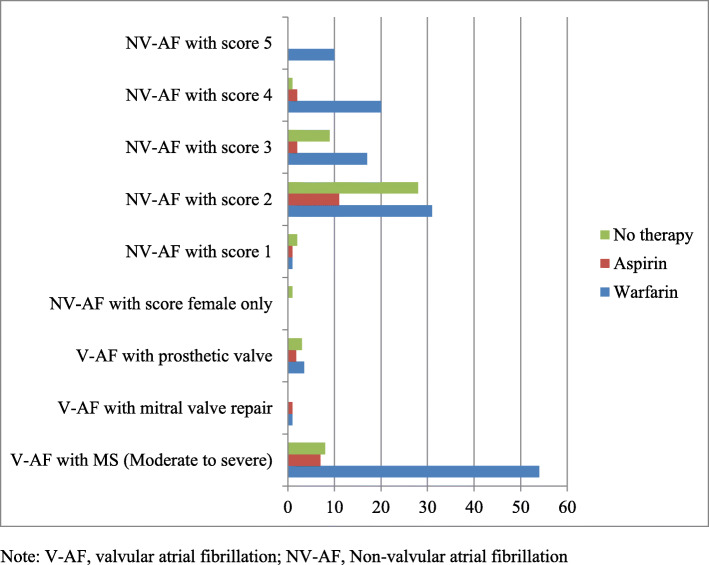
Type of anti-thrombin therapy for valvular AF and non-valvular AF at Cardiac Clinic, UOG hospital, Northwest Ethiopia, December 1, 2018 to September 30, 2019

**Fig. 3:**
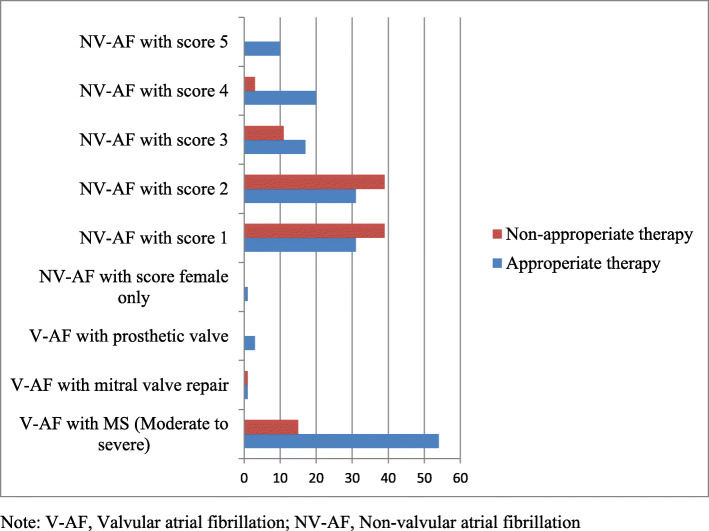
Appropriateness of anti-thrombotic therapy for AF patients at Cardiac Clinic, UOG hospital, Northwest Ethiopia, December 1, 2018 to September 30, 2019

One hundred-sixty-one (76%) patients were on anti-thrombotic therapy, of which 137 (65%) were on warfarin (or warfarin with aspirin), and 24 (11%) were on aspirin. The remaining 49 (24%) were not in any form of anti-thrombotic therapy (Fig. 2). Sixty-six percent (139/210) of study subjects were appropriately treated with anti-thrombotic therapy. Seventy-eight percent (58/74) in valvular AF group and 60% (81/136) in non-valvular AF group were appropriately treated. The rest, 71/210 (34%) AF patients were inappropriately treated. All of the inappropriately treated cases were undertreated or not treated (Fig. 3). The HAS-BLED score was calculated for all patients, and scored 2 or below. Time in INR Therapeutic Range (TTR) was determined for patients on warfarin, and declared to have suboptimal control (TTR = 39.4%).

### Factors associated with appropriate use of anti-thrombotic therapy

Bi-variate and multi-variate logistic regression analyses were used to identify associated factors for appropriate use of anti-thrombotic therapy in patients with AF. On bi-variate analysis, presence of prior stroke/TIA (COR = 3.46, 95% CI: 1.37–8.7, *P* = 0.008), valvular AF type (COR = 3.09, 95% CI: 1.58–6.07, *P* = 0.001) and ‘can afford for regular INR monitoring’ (COR = 8.82 95% CI: 2.80–27.72, *P* < 0.001) were significantly associated with appropriate use of anti-thrombotic therapy. When significant variables in bi-variate analysis were regressed for multi-variate analysis, only ‘can afford for regular INR monitoring’ (AOR = 2.60 95% CI: 1.10–6.10, P = 0.001) was independently associated with appropriate use of guideline directed anti-thrombotic therapy (Table 4).

**Table 4:** Bi-variate and multi-variate logistic regression analysis of patients on anti-thrombotic therapy at Cardiac Clinic, UOG hospital, Northwest Ethiopia, December 1, 2018 to September 30, 2019

Variable	Anti-thrombotic therapy Appro. Inappro.	COR (95%)	P-value	AOR (95%)	*P*-Value
Stroke					
Yes	33 6	3.46 (1.37–8.70)	0.008	2.20 (0.56–8.70)	0.25
No	105 66				
Type of AF					
Valvular	58 16	3.09 (1.58–6.07)	0.001	1.90 (0.80–4.80)	0.14
Non-valvular	81 55				
INR monitoring					
vCan afford	135 56	8.82 (2.80–27.72)	< 0.001	2.60 (1.10–6.10)	0.001
Can’t afford	4 15				
Heart failure					
Yes	124 66	0.8 (0.46–3.40)	0.67		
No	14 6				
Hypertension		0.8 (0.73–3.0)			
Yes	34 13		0.28		
No	104 59				
Thyrotoxicosis					
Yes	19 19	0.45 (0.22–0.90)	0.026	0.80 (0.40–1.90)	0.67
No	119 53				
Age (years)					
≥ 75	15 3	2.80 (0.78–10.03)	0.13	2.20 (0.50–8.70)	0.25
< 75	123 69				
Place of Residence					
Urban	68 34	1.08 (0.50–1.60)	0.77		
Rural	70 38				
Formal education					
Yes	101 56	0.78 (0.60–2.50)	0.41		
No	37 16				

Note: *AF* atrial fibrillation, *COR* crude odds ratio, *AOR* adjusted odds ratio

## Discussion

A total of 210 patients with atrial fibrillation were included in the study. Seventy-four (35%) had valvular AF, while 136 (65%) had non-valvular AF. Although rheumatic heart disease remained to be a major risk factor for AF in sub-Saharan Africa, non-valvular cardiac causes are evident escalating health problems. Economic and social changes in sub-Saharan Africa are driving on epidemiologic transition to a double burden of disease in the region [4–7].

Heart failure (91%) was the commonest identified clinical condition in AF patients, followed by hypertension (22%), stroke/TIA (19%), and hyperthyroidism (18%). Valvular heart disease was detected in half (52%) of heart failure cases. Almost all Patients with AF presented late with symptomatic structural heart disease. Rheumatic heart disease was documented as the prevalent cause of valvular heart disease in sub-Saharan Africa [5–8, 18, 19]. Western world studies documented hypertension (30–70%), coronary artery disease (20–40%) and diabetes (15–25%) were common causes of AF; while valvular heart disease (30–60%), hypertension (30–40%), dilated cardiomyopathy (5–15%) and coronary artery disease (5–10%) were reported AF causes in sub-Saharan Africa [6–8, 11, 18]. This clinical data demonstrated some shared risk factors for AF among developed and developing countries.

Among 210 study subjects, 139 (66%) subjects were appropriately treated with anti-thrombotic therapy. Appropriately treated subjects in valvular AF group and non-valvular AF group were 58/74 (78%) and 81/136 (60%) respectively. Possible explanation for difference in magnitude of appropriately treated subjects among AF groups might be better physicians’ experience in managing valvular AF patients, or non-valvular AF patients required arithmetic calculation of CHA_2_DS_2_VASc risk score.

Study-based estimated anti-thrombotic utilization rate among eligible AF patients were 70–85% in Europe, 50–60% in Asia and 30–60% in Sub-Saharan Africa. Utilization rate in this study was 66%, higher than a few African study reports. Variable utilization rate in anti-thrombotic therapy among nations might be related to differences in standard health care and socio-economic conditions [3, 5, 6, 10–14, 19, 20].

This study showed 39.4% patients on warfarin had good INR control (INR = 2–3), within range of other African study reports. Global reports on therapeutic INR control were 70–80% in Europe, 40–60% in Asia, and 30–50% in Africa [4, 6, 8, 19]. Lack of proper titration of anticoagulant dosage was fundamental reason for sub-therapeutic INR values. Quality of anticoagulation with warfarin is reflected by Time in INR Therapeutic Range (TTR), and patients on warfarin should achieve their TTR ≥ 65% of the follow-up time. This study revealed calculated HAS-BLED score was 2 or below for all patients. HAS-BLED score is used to assess the risk of bleeding, in which high risk patients (score ≥ 3) required frequent review and follow-ups [21].

In bi-variate analysis, odds of appropriate therapy were provided three fold higher to valvular AF patients (COR = 3.09, 95% CI: 1.58–6.07, *P* = 0.001) as compared with non-valvular AF patients. Similarly, patients with prior stroke/TIA were three fold higher (COR = 3.46, 95% CI: 1.37–8.7, *P* = 0.008) appropriately treated as compared with those without stroke/TIA. Explanatory reasons could be patients with valvular AF or prior stroke/TIA were ‘high risk’ patients, in whom anti-thrombotic therapies were directly indicated.

In multi-variate analysis, patients who ‘can afford for regular INR monitoring’ were two and half fold higher (AOR = 2.60 95% CI: 1.10–6.10, *P* = 0.001) appropriately treated as compared with those who ‘can’t afford for regular INR monitoring’. This finding was congruent with studies in Africa and Asia in which underutilization of anti-thrombotic therapy were reported among those who had financial constraints [5–7, 14]. Physicians in the setting prescribe and adjust dosage of warfarin, when AF patients have recent INR determination at hand.

Novel oral anticoagulants (NOACs) are recently introduced anticoagulants, which have a number of advantages over warfarin despite high cost and limited access to antidote (reversal agent). NOACs have more rapid onset and offset than warfarin, are prescribed in fixed doses, have fewer documented food and drug interactions, and do not require routine anticoagulant monitoring. Their use might be considered for eligible non-valvular AF patients who had limited access to INR monitoring [5, 14, 20].

### Limitation of the study

Identified associated factors might not be causal for appropriate use of antithrombotic therapy in cross-sectional study. Selection bias couldn’t be avoided as consecutive sampling method was used to recruit study subjects.

## Conclusions

Non-valvular AF constitutes two-thirds as cause of AF in the setting, prevailing epidemiologic transition to non-communicable diseases. Sixty-six percent of AF patients eligible for anti-thrombotic therapy were appropriately treated. ‘Can afford for regular INR monitoring’ was independently associated with appropriate use of anti-thrombotic therapy.

### Recommendation

The authors recommend large scale longitudinal study to improve understanding of AF in Ethiopia. Intervention program to access ‘regular INR monitoring’ should be practiced to escalate utilization rate of anti-thrombotic therapy (warfarin) in eligible AF patients.

## Supplementary information

**Additional file 1.** Questionnaire.

## Data Availability

All data generated and analyzed are included in this research article.

## Abbreviations

AF: Atrial fibrillation
AHA: American Heart Association
ACC: American college of cardiology
AOR: Adjusted odds ratio
CI: Confidence interval
CHA_2_DS_2_VASc score: Congestive heart failure, hypertension, age ≥ 75, diabetes, stroke or TIA, vascular disease (prior myocardial infarction, peripheral arterial disease or aortic plaque), and sex category (female)
ECG: Electrocardiography
ECHO: Echocardiography
HRS: Heart Rhythm Society
HAS-BLED score: Uncontrolled hypertension, abnormal liver and kidney disease, stroke, bleeding predisposition or tendency, labile INR, elderly (age ≥ 65 years), and drugs or alcohol
INR: International normalized ratio
MI: Myocardial infarction
NOACs: Novel oral anticoagulants
PAD: Peripheral arterial disease
TIA: Transient ischemic attack
TSH: Thyroid stimulating hormone
TTR: Time in INR therapeutic range
VKA: Vitamin K antagonists
