# Supplementary material for: Appropriate use of anti-thrombotic therapy in patients with atrial fibrillation at single-center experience, Northwest Ethiopia
Source: BMC Cardiovasc Disord. 2020 Aug 17;20:375. doi: 10.1186/s12872-020-01659-y (PMC7433135; doi:10.1186/s12872-020-01659-y)
Supplement: Supplementary file 1 — Additional file 1. Questionnaire. [file 12872_2020_1659_MOESM1_ESM.docx]

**Questionnaire (English version)**

Appropriate use of antithrombotic therapy in patients with atrial fibrillation in University of Gondar Hospital, Gondar, Northwest Ethiopia

**Section 1: Socio-demographic data**

1. Date of data collection __________
2. Chart Number
3. Age (in years)
4. Sex A. male B. female
5. Residence A. Urban B. rural
6. Marital status A. Married B. single C. divorced D. widowed
7. Occupation
8. Farmer
9. Merchant
10. Government employee
11. Housewife
12. Student
13. daily laborer
14. others (specify)
15. Religion
16. Orthodox Christian
17. Protestant
18. Catholic
19. Muslim
20. Other(specify)
21. Level of education
22. Cannot read and write
23. Read and write only
24. Completed primary school
25. Completed secondary school
26. Diploma and above
27. Monthly income ( in birr)
28. <1500 B. 1500-3000 C.>3000

**Section 2: Risk factor identification and other clinical data**

1. Smoking status
2. yes(current, mention in pack-years)
3. yes(past)
4. never
5. Do you drink alcohol?
6. Never
7. Occasionally
8. Usually(quantify)
9. Pulse(irregularly irregular)
10. Yes
11. No
12. BP(mention if on anti-hypertensive medication)----------------
13. Anthropometry
14. Weight
15. Height
16. BMI(body mass index)
17. History of heart failure
18. Yes
19. No
20. Diabetes mellitus
21. Yes
22. No
23. History of stroke or TIA(transient ischemic attack)
24. Yes
25. No
26. If yes to number 8, was he/ she on antithrombotic therapy before the event of stroke or TIA
27. Yes(specify the antithrombotic)
28. No
29. If he/she was on warfarin(for question no 9) what was the recent INR value before stroke/TIA-------
30. Thyrotoxicosis
31. Yes
32. No
33. Chronic kidney disease
34. Yes(recent serum creatinine)
35. No
36. History of obstructive sleep apnea
37. Yes
38. No
39. ECHO findings(conclusion)
40. CRVHD(mention specific valve lesions)
41. IHD
42. DCMP
43. HHD
44. DVHD
45. Others(specify)--------
46. Peripheral arterial disease(if Doppler US done)
47. Yes
48. No
49. ECG-conclusion of findings…………
50. Lipid profile(if available)
51. Does the patient have Prosthetic valves
52. Yes (mention the type )
53. Date of AF diagnosis(from the chart)-------
54. No

**Section 3: management of the patient and assessing the risk of bleeding**

1. Is the patient on rate control therapy
2. yes(specify)
3. No
4. Is the patient on antithrombotic therapy?
5. Yes
6. No
7. If yes for no. 21 ,which anticoagulant
8. Warfarin
9. Aspirin
10. DOAs(specify)
11. If aspirin is used, why?(please ask the patient and check for documentation by physicians)
12. Patient cannot afford for follow up INR
13. Patient preference
14. Patient not informed
15. Because of side effects to other anticoagulants (specify)
16. Because of contraindications to other antithrombotics (specify)
17. IF NO for question no. 21, why? (please check for choice **B** and **D** only; others will be answered by question number 22)
18. Not eligible to start
19. Patient refuse to take
20. Because of Contraindications
21. Patient not informed about it
22. Because of medication side effects
23. Risk of bleeding from anticoagulant therapy and contraindication to OACs(circle it if applicable)
24. Advanced age (65-75 yrs/>75 yrs)
25. Uncontrolled hypertension
26. Renal disease (mention Cr.)
27. Liver disease(mention if LFT or US results are available)
28. History of stroke
29. Prior bleeding or predisposition to bleeding(like the use of NSAIDs)
30. Platelet count
31. INR(if available)
32. Pregnancy
33. Previous history of hypersensitivity to OACs or bleeding after the use of OACs
34. Recent INR value(if on warfarin therapy) ----------------
